# Microhardness and Abrasive Wear-Induced Material Loss in FDM-Printed PLA, PETG, ABS, ASA, and Fe-Filled PLA

**DOI:** 10.3390/polym18212620

**Published:** 2026-10-22

**Authors:** Mustafa Tekin, Şükrü Kaya, Görkem Yumuşak

**Affiliations:** 1 Department of Materials Science and Nanotechnology Engineering, Faculty of Engineering and Natural Sciences, Yeditepe University, 34755 Istanbul, Türkiye; 2 Center for Nanotechnology and Biomaterials Applications and Research (NBUAM), Marmara University, 34854 Istanbul, Türkiye; s.kaya@beu.edu.tr; 3 Department of Mechanical Engineering, Faculty of Engineering and Architecture, Bitlis Eren University, Rahva Campus, 13100 Bitlis, Türkiye; 4 Department of Metallurgical and Materials Engineering, Faculty of Engineering, Marmara University, Recep Tayyip Erdoğan Campus, M3 Building, 34854 Istanbul, Türkiye; gorkem.yumusak@marmara.edu.tr

**Keywords:** fused deposition model, additive manufacturing, abrasive wear-induced material loss, polymer tribology, microhardness, Fe-filled PLA, surface morphology

## Abstract

This exploratory study comparatively evaluated the time-dependent abrasive wear-induced material loss, local microhardness, and worn-surface morphology of fused deposition modeling (FDM)-printed PLA, PETG, ABS, ASA, and PLA containing 1 wt.% Fe under controlled wet–abrasive grinding. Two physical cylindrical specimens from the same print build were tested concurrently in opposing holder positions against P180 SiC abrasive paper under continuous water lubrication for 60 min at a normal load of 5 N. After 60 min, cumulative mass losses were 11.1% for PLA, 13.0% for PETG, 22.6% for PLA–1 wt.% Fe, 39.3% for ABS, and 50.1% for ASA. Based on the two specimen-level means, Vickers microhardness (HV0.1) was highest for PLA–1 wt.% Fe (28.5), followed by PLA (22.4) and ABS (13.3), while ASA (11.9) and PETG (11.8) showed very similar values. Optical microscopy and selected SEM/EDS observations revealed material-dependent surface features and showed local Fe detectability in the analyzed regions of the filled PLA. The microhardness ranking did not follow the cumulative material-loss trend, most clearly for Fe-filled PLA and for PETG versus ASA. These results indicate that the local indentation-hardness ranking did not correspond directly to the cumulative mass-loss ranking of the investigated FDM-printed polymers under the selected conditions. For material selection in FDM components subjected to abrasive contact, these findings indicate that local microhardness should not be used alone as a proxy for condition-specific abrasive wear performance.

## 1. Introduction

Additive manufacturing has become increasingly important, not only for rapid prototyping but also for the production of functional end-use components [1,2]. Among the available additive manufacturing methods, fused deposition modeling has attracted particular attention because of its simplicity, relatively low cost, and wide material availability. In this technique, the physical and mechanical characteristics of the filament strongly affect both the printing quality and the service performance of the final part. For this reason, material selection is a critical step, especially in applications where printed components are exposed to repeated contact, friction, or surface damage during use. PLA, ABS, ASA, and PETG are among the most widely used filament types, and each of them offers a different balance of stiffness, toughness, printability, and environmental resistance [3,4].

PLA is one of the most widely used materials in fused deposition modeling because it is easy to print, shows good dimensional stability during fabrication, and is derived from renewable resources [5,6]. Its relatively high stiffness is advantageous for many applications, but its limited thermal resistance and brittle behavior may restrict its performance under demanding service conditions [6]. In addition, previous studies have shown that the mechanical performance of printed PLA is highly sensitive to production parameters, which further supports the need for material-specific evaluation under controlled laboratory conditions [7]. To improve its properties, different reinforcement strategies have been explored, including the incorporation of natural fibers and metallic fillers [8,9]. Metal-containing PLA systems are of particular interest because they may alter hardness and mechanical response. However, the final behavior of such materials depends not only on the presence of the filler, but also on how well it is distributed within the polymer. For this reason, PLA containing 1 wt.% Fe was included in the present study as an additional material for comparative material-loss evaluation. A laboratory-scale version of this PLA-based composite filament, together with its production route and preliminary mechanical performance, was presented previously in a conference proceeding [10]. A low Fe content of 1 wt.% was selected to preserve printability and filament continuity while introducing detectable metallic filler content. Because the neat PLA and PLA–1 wt.% Fe filaments were produced through different routes, the present study was not designed to isolate the independent effect of Fe addition; the Fe-containing filament was therefore treated as a distinct material formulation rather than as a controlled Fe-modified counterpart of the commercial PLA. A matched laboratory-produced neat PLA control processed through the same filament-production route was not included in the original experimental campaign; consequently, the present dataset does not permit the specific contribution of Fe addition to be separated from effects associated with filament-production history.

ABS is widely used in fused deposition modeling because of its good balance of toughness, impact resistance, and thermal stability. At the same time, its tendency to shrink and warp during printing can make dimensional control more difficult [11]. ASA is generally considered an alternative to ABS, especially for outdoor applications, because it offers similar general mechanical behavior, together with better resistance to weathering and ultraviolet exposure [12]. PETG is often preferred because it combines relatively easy printability with good impact resistance and low shrinkage during printing [13]. Because these materials differ in both mechanical response and service characteristics, their wear behavior under abrasive conditions may also differ significantly. From a sustainability perspective, durability under abrasive contact is also relevant to material selection for additively manufactured components. From this perspective, comparative material-loss data obtained under a defined abrasive condition may provide one laboratory-scale consideration for durability-oriented material selection, although such results should not be directly interpreted as service-life or sustainability performance.

Previous studies have examined the tribological behavior of FDM polymers under substantially different experimental configurations. For example, PETG has been investigated under dry sliding conditions [14], PLA and ABS have been compared under sliding-wear configurations [15], the tribological response of ASA has been evaluated as a function of printing parameters [16], and PLA, ABS, and HIPS have been studied using ball-on-disc testing with different layer thicknesses [17]. Classical polymer tribology has long indicated that abrasive wear cannot be interpreted from hardness alone. Ratner et al. related the abrasion resistance of polymers to a combination of mechanical properties [18], while Lancaster subsequently reported an approximately linear relationship between abrasive wear rate and 1/(Sε), where S is the breaking strength and ε is the elongation to break [19]. Accordingly, the novelty of the present study does not lie in the general observation that hardness alone is insufficient to predict polymer wear, but in the condition-specific side-by-side comparison of five FDM filament systems under the same wet fixed–abrasive grinding protocol, together with time-resolved gravimetric, local microhardness, and surface-characterization data. More recently, Kujawa and Ptak [20] compared the abrasion resistance of several FDM-printed and conventionally extruded polymers and reported that hardness did not show a simple relationship with abrasion resistance. Collectively, these studies demonstrate the strong dependence of polymer wear behavior on material, printing conditions, and test configuration.

Accordingly, the specific knowledge gap addressed in this study is the limited availability of side-by-side data for widely used FDM polymers evaluated under the same wet fixed–abrasive grinding condition together with local microhardness and worn-surface characterization. The scientific contribution of the present work therefore lies in providing a controlled, condition-specific comparison of PLA, PETG, ABS, ASA, and a laboratory-produced PLA–1 wt.% Fe formulation using the same specimen geometry, abrasive grade, nominal load, lubrication condition, rotational configuration, and measurement schedule. Percentage mass loss was used as the primary response, while optical microscopy, selected SEM/EDS observations, and Vickers microhardness measurements provided complementary information on material response. Accordingly, the main research question was whether the material ranking based on local microhardness corresponded to the cumulative mass-loss ranking of the investigated FDM polymers under the same wet fixed–abrasive grinding condition. The study is not intended to establish a universal hardness–wear relationship or a universal ranking of FDM polymers, but rather to provide a controlled descriptive comparison under a clearly defined abrasive condition.

## 2. Materials and Methods

### 2.1. Materials and Specimen Preparation

Commercial PLA, ABS, ASA, and PETG filaments with a nominal diameter of 1.75 mm were used in this study. All commercial filaments were supplied by Fibromast, Istanbul, Türkiye. In addition to these materials, a laboratory-produced PLA filament formulated with a nominal Fe content of 1 wt.% was included as an Fe-filled PLA formulation. The designation PLA–1 wt.% Fe refers to the nominal Fe loading used during filament preparation and should not be interpreted as a bulk Fe concentration independently verified after filament production. The Fe particles used in the PLA matrix had a reported purity of 99% and a particle-size range of 50–100 nm and were obtained from existing laboratory stock. Further details regarding the filament-preparation route and its preliminary mechanical characterization were reported previously [10].

All specimens were fabricated using a Creality K1 FDM printer (Shenzhen Creality 3D Technology Co., Ltd., Shenzhen, China) equipped with a 0.4 mm nozzle. To provide a consistent basis for comparison, all specimens were produced with the same nominal cylindrical geometry and the same principal printing strategy. The specimens had a diameter of 30 mm and a height of 20 mm. The same build orientation was used for all material groups, and the flat circular base surface of each cylinder was subjected to wet–abrasive grinding. The principal printing parameters were maintained as consistently as possible, while only the material-specific nozzle and bed temperatures were adjusted within the recommended processing ranges to obtain printable specimens without visible macroscopic defects. All specimens were printed using 100% nominal infill, a concentric infill pattern, and one wall. Specimens showing visible defects, including under-extrusion, layer separation, or warpage, were excluded. The main printing parameters are summarized in Table 1.

### 2.2. Wet–Abrasive Grinding Procedure

Wet–abrasive grinding experiments were conducted using a semi-automatic metallographic grinding and polishing system (RotoPol 22) equipped with a specimen head (RotoForce 4, Struers ApS, Ballerup, Denmark). P180 SiC abrasive paper was fixed to the rotating platen, and water was supplied continuously as the lubricating medium. The platen speed was set to 300 rpm, while the specimen-head speed was set to 150 rpm in co-rotation mode. A nominal normal load of 5 N was applied to each specimen, and each test was continued for 60 min. Based on the nominal circular contact area of 706.9 mm^2^, the applied load corresponded to a nominal average contact pressure of approximately 7.1 kPa. This value assumes complete and uniform contact over the circular specimen base and does not represent local contact-pressure variations. Because material removal progressively altered the specimen geometry and effective contact condition, particularly for the higher-loss materials, this nominal pressure was used only to describe the initial loading condition and was not assumed to remain representative throughout the 60 min test.

The test parameters were selected to provide a consistent laboratory-scale abrasive condition for all materials and to obtain measurable mass loss over the test period. P180 SiC paper was chosen as a relatively coarse fixed abrasive. Water was supplied continuously to maintain a wet grinding condition, remove loose debris, and limit excessive frictional heating. These conditions were selected for comparative laboratory testing and were not intended to reproduce a specific standardized wear test or service condition.

Accordingly, the present test represents a water-lubricated fixed–abrasive grinding condition in which the full circular specimen face is pressed against P180 SiC paper while both the platen and specimen head rotate. Because the platen and specimen head co-rotated at different rotational speeds, the local relative sliding velocity varied across the full-face contact, and a single representative sliding distance could not be defined reliably for the test. Because the contact geometry, kinematics, abrasive medium, and loading arrangement differ from standardized abrasive-wear methods, the measured responses are reported as condition-specific comparative mass loss rather than as standardized wear coefficients or universally transferable wear properties.

**Figure 1:**
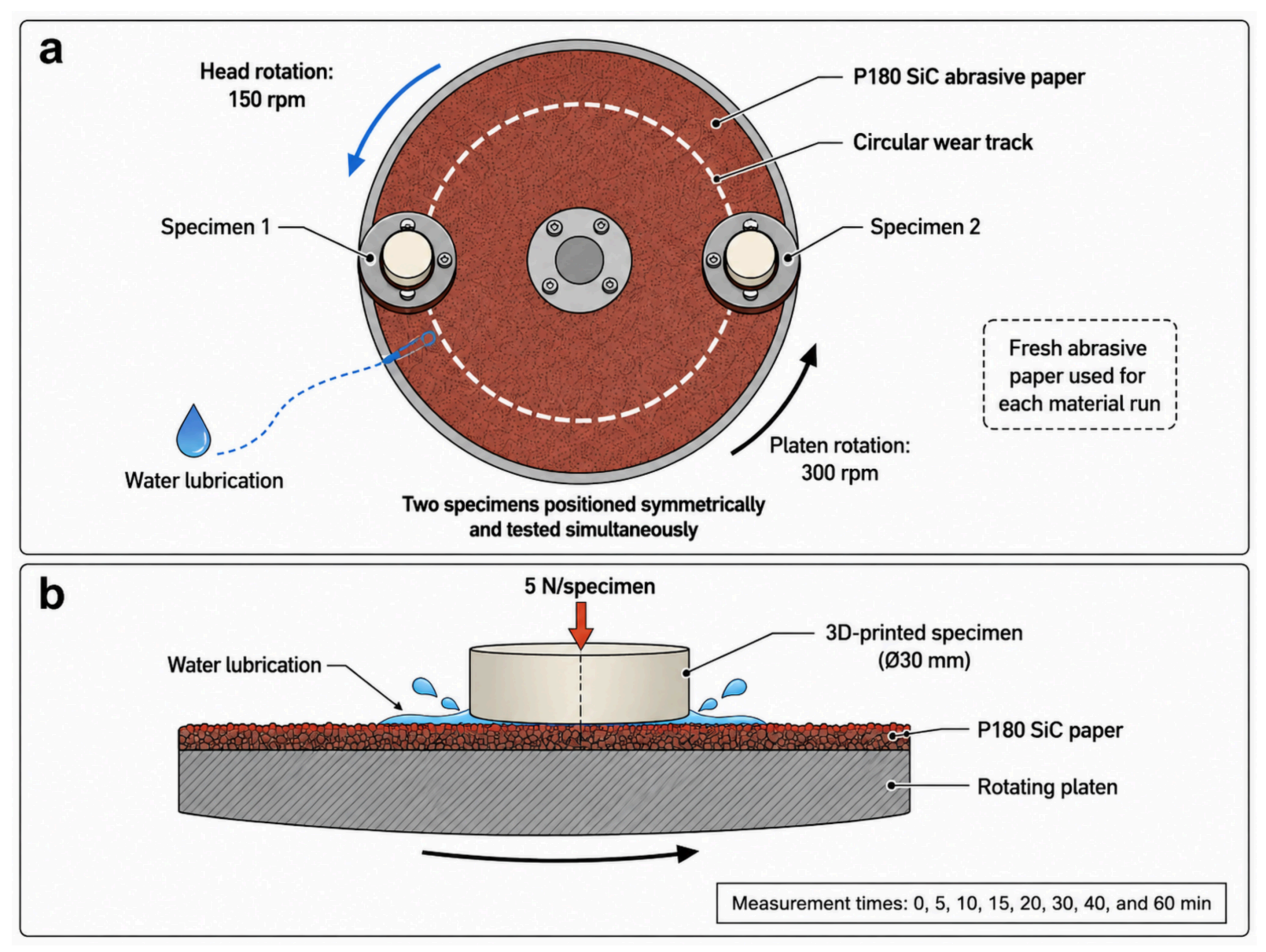
Schematic representation of the laboratory-scale wet–abrasive grinding configuration: (**a**) top view showing the symmetric positioning of two specimens on the rotating P180 SiC abrasive paper under continuous water lubrication; (**b**) cross-sectional view showing the specimen–abrasive contact and the nominal normal load of 5 N applied to each specimen. The platen and specimen head rotated at 300 and 150 rpm, respectively. The specimen diameter was 30 mm, and mass measurements were performed at 0, 5, 10, 15, 20, 30, 40, and 60 min. The schematic illustration was created with the assistance of ChatGPT (GPT-5.6 Sol, OpenAI) and subsequently reviewed and edited by the authors.

Two physical specimens from the same print build were mounted simultaneously in two fixed diametrically opposed specimen-holder positions. Since tangential velocity in a rotary grinding system depends on radial position, all specimens were placed at the same fixed nominal position on the SiC abrasive paper. A fresh sheet of P180 SiC abrasive paper was installed at the beginning of each two-specimen run. The same paper remained in use throughout the complete 60 min sequence and was replaced before testing the next material group. A schematic representation of the wet–abrasive grinding configuration and specimen loading arrangement is shown in Figure 1.

Each 60 min experiment consisted of seven sequential grinding intervals: four 5 min intervals, two 10 min intervals, and one final 20 min interval. At the end of each interval, the specimens were removed, rinsed to remove loosely attached debris, dried using an air jet, and weighed using an analytical balance with a readability of 0.0001 g. The same rinsing and air-drying procedure was applied before each mass measurement. Two physical specimens from the same print build were available for each material group and were ground simultaneously in opposing holder positions during the same material run. Thus, the dataset includes two physical specimens per material but does not provide independent build-to-build or run-to-run replication. Given this limited replication structure, the abrasive-grinding dataset was treated as exploratory and descriptive. The reported means and standard deviations are used to describe the variability between the two physical specimens, while the lack of independent build-to-build and run-to-run replication is explicitly acknowledged.

The material-loss response was evaluated using cumulative percentage mass loss relative to the initial mass of each individual specimen. Cumulative percentage mass loss at each time point was calculated as:

Cumulative mass loss (%) = [(m_0_ − m_t_)/m_0_] × 100 (1)

where m_0_ is the initial specimen mass and m_t_ is the specimen mass measured after the corresponding grinding duration. Material-loss rates were calculated by dividing the change in cumulative percentage mass loss by the corresponding time interval. In addition to the broader 0–20 min and 20–60 min periods, interval-based rates were also determined for each successive measurement interval (0–5, 5–10, 10–15, 15–20, 20–30, 30–40, and 40–60 min). All rates were expressed as percentage points per minute.

Percentage mass loss was retained as the primary comparative response. The post-test apparent bulk density of the ground specimens was measured using the Archimedes principle. These values were used to estimate the specimen-level volumetric material loss reported in the Supplementary Materials. A specific wear rate was also not reported because a representative sliding distance could not be reliably determined for the present co-rotating platen and specimen-head configuration.

### 2.3. Microhardness and Surface Characterization

Vickers microhardness measurements were performed using an EMCO-TEST DuraScan 20 hardness tester (EMCO-TEST Prüfmaschinen GmbH, Kuchl, Austria) on the unworn rear surfaces of both specimens used in the grinding experiment for each material, using a common test load of HV0.1 and a dwell time of 15 s. Vickers microindentation was employed as a complementary local surface-response measurement rather than as a standardized bulk hardness test for plastics. The method was selected to compare the local indentation response of all investigated materials using the same test force, dwell time, and surface-preparation procedure; consequently, the reported HV0.1 values should not be interpreted as equivalent to hardness values obtained using ASTM D785 or ASTM D2240. Before indentation, the rear surfaces were lightly ground using P1000 SiC abrasive paper to remove the as-printed surface texture of the last-deposited layer and to obtain a sufficiently flat measurement surface. The unworn rear surface was selected to provide a sufficiently flat and consistently prepared surface for indentation measurements. Five indentations were made at spatially separated locations on each of the two grinding specimens per material, while avoiding specimen edges, visible surface defects, and previously indented regions. Both specimens were indented under identical conditions and after the same surface preparation. The two specimens are reported separately and were not pooled into a single sample; the reported mean and standard deviation therefore represent within-specimen variation among the five local indentations of each specimen, while the difference between the two specimen means provides the specimen-to-specimen comparison within the print build. The microhardness results were evaluated descriptively, and no inferential statistical analysis was applied. Following the grinding experiments, the worn surfaces of all material groups were examined using a Nikon ECLIPSE L150 optical microscope (Nikon Corporation, Tokyo, Japan) at 40× magnification.

Selected neat PLA and PLA–1 wt.% Fe surfaces were further examined using a ZEISS EVO 10 scanning electron microscope (Carl Zeiss Microscopy GmbH, Jena, Germany). Prior to SEM examination, the specimens were sputter-coated with a thin Au layer to provide electrical conductivity. The selected SEM images were acquired using secondary-electron imaging at accelerating voltages of 10–20 kV. Because the acquisition settings differed among the selected images, the morphological observations were interpreted qualitatively rather than used for quantitative image-to-image comparison. SEM observations were performed on the worn surfaces of neat PLA and PLA–1 wt.% Fe, together with the unworn rear surface of PLA–1 wt.% Fe. Energy-dispersive X-ray spectroscopy was conducted using a Bruker XFlash Detector 730M-250 (Bruker Nano GmbH, Berlin, Germany) on selected regions of the worn and unworn PLA–1 wt.% Fe surfaces to evaluate the local detectability of Fe. The SEM/EDS findings were interpreted as complementary surface-level observations limited to the analyzed regions. No bulk elemental analysis, systematic multi-region mapping, or quantitative particle-dispersion analysis was available; therefore, the EDS measurements were not used to verify the nominal bulk Fe content or to quantify filler dispersion or agglomeration.

### 2.4. Generative AI Use

Generative AI (ChatGPT, GPT-5.6 Sol, OpenAI) was used for language editing, readability improvement, manuscript organization, and assistance in preparing the schematic illustration presented in Figure 1 and the graphical abstract. All AI-assisted outputs were reviewed and edited by the authors, who take full responsibility for the content of the manuscript. Generative AI was not used to generate, modify, analyze, or interpret primary experimental data.

## 3. Results and Discussion

### 3.1. Time-Dependent Material Loss Under Wet–Abrasive Grinding

**Figure 2:**
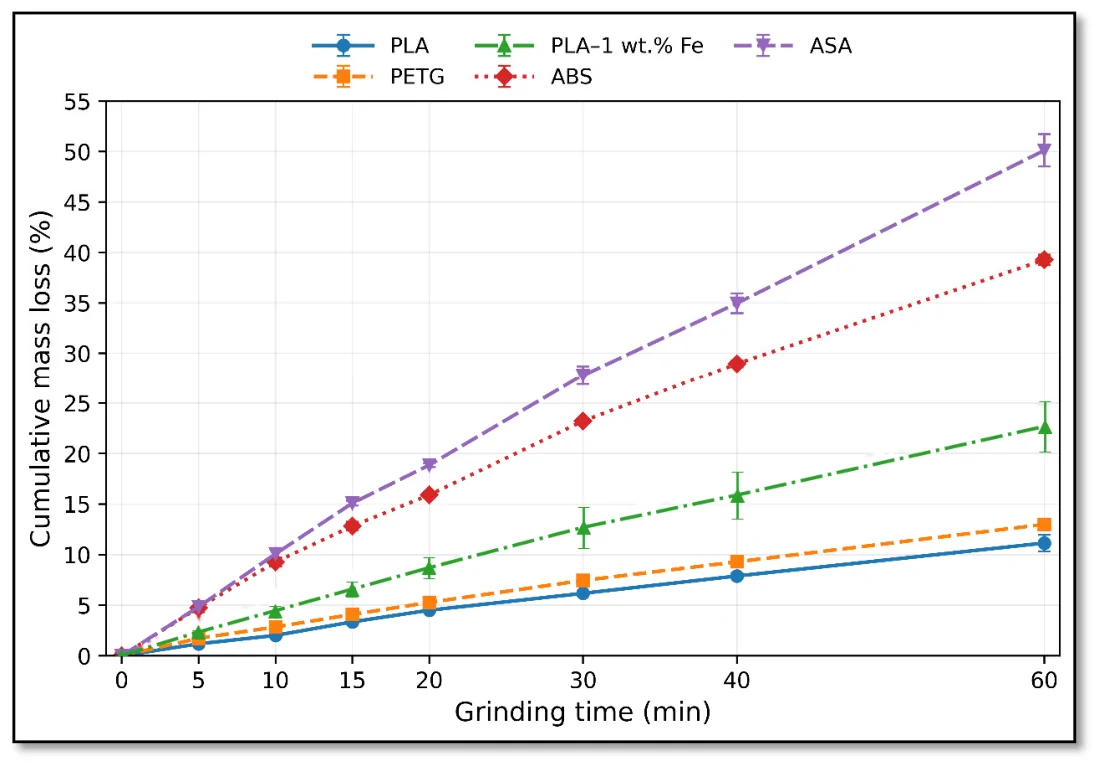
Time-dependent cumulative mass loss of the investigated FDM filaments during the 60 min wet–abrasive grinding procedure. Data are presented as the descriptive mean ± standard deviation of two physical specimens from the same print build tested concurrently during the same material run.

Figure 2 presents the time-dependent cumulative mass loss of the investigated filament groups during the 60 min wet–abrasive grinding procedure. Cumulative loss was calculated relative to the individual initial mass of each specimen. Because the two specimens within each material originated from the same print build and were tested concurrently within the same material run, the results are presently interpreted as exploratory descriptive comparisons rather than as evidence of build-to-build or run-to-run reproducibility.

The cumulative material-loss curves showed condition-specific separation in the descriptive mean responses of the investigated filament groups. After 60 min, the descriptive mean losses were 11.15 ± 0.81% for PLA, 13.00 ± 0.46% for PETG, 22.62 ± 2.51% for PLA–1 wt.% Fe, 39.27 ± 0.51% for ABS, and 50.12 ± 1.60% for ASA. PLA and PETG showed the lowest observed descriptive mean losses throughout the procedure, whereas ABS and ASA showed higher descriptive mean material removal under the investigated conditions. The PLA–1 wt.% Fe formulation displayed an intermediate response, with greater material loss than neat PLA and PETG but lower material loss than ABS and ASA. 

The separation among the material groups developed progressively with grinding time. At 20 min, the descriptive mean cumulative losses were approximately 4.50% for PLA, 5.28% for PETG, 8.66% for PLA–1 wt.% Fe, 15.90% for ABS, and 18.86% for ASA. The same general ordering was maintained during the later stages of the procedure.

**Figure 3:**
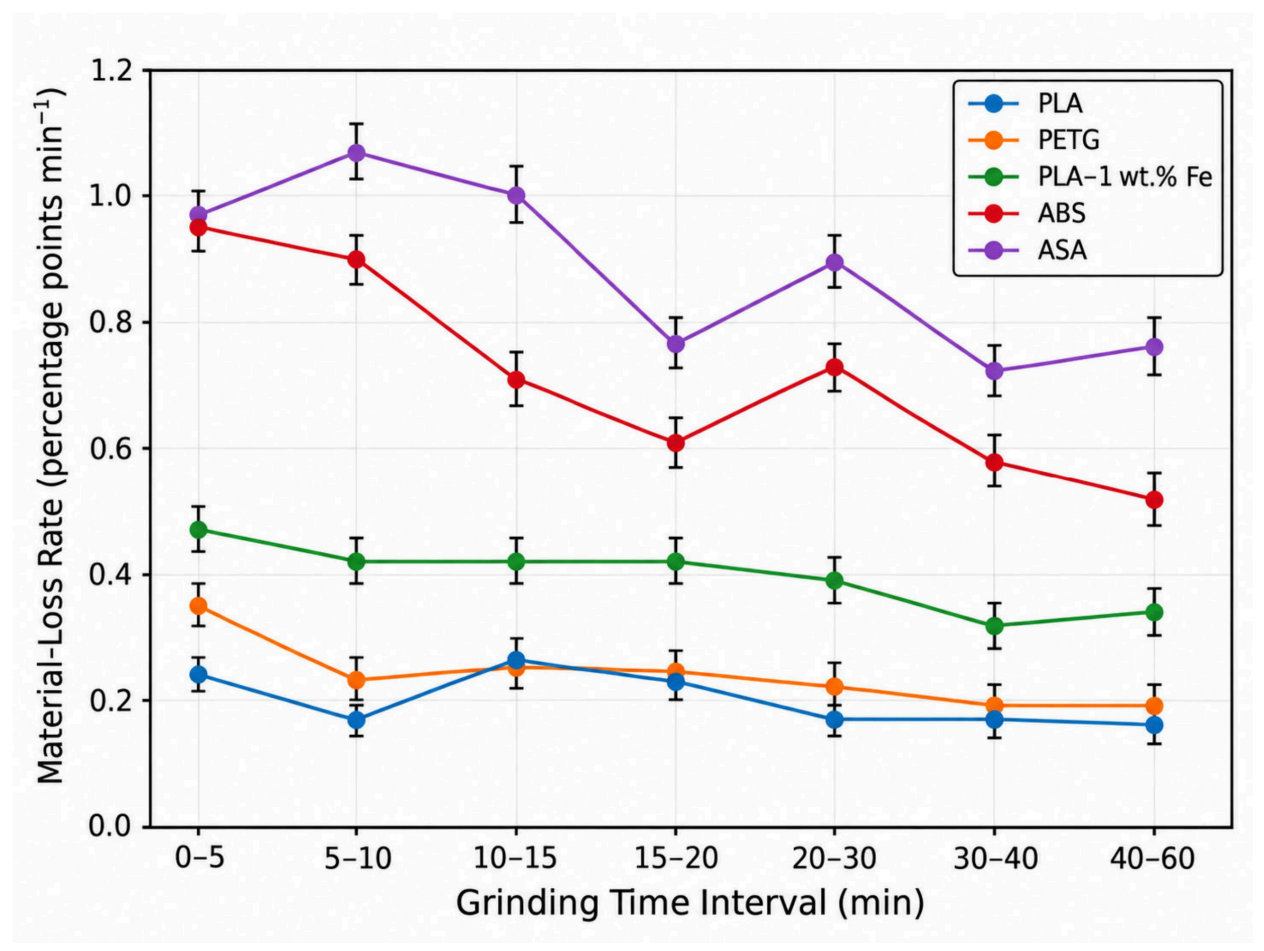
Interval-based material-loss rates of PLA, ASA, PETG, ABS, and PLA–1 wt.% Fe during successive stages of the 60 min wet–abrasive grinding test. The rates were calculated from the change in cumulative percentage mass loss between consecutive measurement points divided by the corresponding time interval. Curves represent the descriptive mean values of the two concurrently tested specimens, and error bars indicate the standard deviation between the two specimen-level interval rates. The curves and error bars are presented for descriptive comparison of the interval-dependent trends.

To examine the time dependence of material removal in greater detail, interval-based material-loss rates were calculated for each successive measurement period and are presented in Figure 3. The plotted trends show that ASA and ABS generally exhibited the highest material-loss rates throughout the test, whereas PLA and PETG maintained the lowest rates. PLA–1 wt.% Fe displayed an intermediate response, with higher rates than neat PLA and PETG but lower rates than ASA and ABS. In general, the interval-based rate curves indicate that material removal tended to slow with increasing grinding time, although some fluctuations were observed between consecutive intervals. These fluctuations may reflect the combined effects of evolving specimen-surface conditions, changes in abrasive cutting efficiency, debris accumulation, and the repeated interruption–rinsing–drying–reloading sequence between measurement intervals. Progressive material removal may also have altered the specimen geometry and effective contact area, particularly for the higher-loss ABS and ASA specimens; consequently, the nominal contact pressure may have decreased during the later stages of grinding and may have contributed to the observed reduction in material-loss rate. When the data were considered over broader periods, the average material-loss rates during the initial 0–20 min period were approximately 0.23, 0.26, 0.43, 0.80, and 0.94 percentage points per minute for PLA, PETG, PLA–1 wt.% Fe, ABS, and ASA, respectively. During the subsequent 20–60 min period, the corresponding rates decreased to approximately 0.17, 0.19, 0.35, 0.58, and 0.78 percentage points per minute. Thus, all material groups showed lower average material-loss rates during the later stage of the test, although the magnitude of this reduction differed among materials.

The lower average rates observed during the later period, together with the fluctuations seen in the interval-based curves, were not interpreted as evidence of a uniquely defined steady-state wear regime. The observed change may reflect the combined effects of evolving specimen-surface conditions, debris accumulation, and progressive reduction in the cutting efficiency of the SiC abrasive paper, which remained in use throughout each individual 60 min run. Therefore, the curves are interpreted as descriptive material-loss trends specific to the selected laboratory-scale grinding protocol. Because the condition of the abrasive paper was not independently quantified during the run, the contribution of abrasive wear, clogging, and debris accumulation cannot be separated from changes in the response of the polymer specimens. Accordingly, the reduction in material-loss rate with time should not be attributed solely to evolution of the polymer surface. Continuous water lubrication was used throughout the test and may have moderated frictional heating; however, the specimen–abrasive interface temperature was not measured. Therefore, localized thermally assisted softening or smearing cannot be excluded as a contributing factor to the observed material-removal response, although its contribution cannot be separated from mechanically induced deformation using the present dataset.

Table 2 summarizes the initial and final specimen masses together with the corresponding cumulative percentage losses. The two PLA–1 wt.% Fe specimens showed greater variation in their initial masses than the other material groups. Percentage loss was therefore calculated separately for each specimen relative to its own initial mass before the descriptive group mean was obtained. Thus, the reported percentage-loss values were not calculated from pooled group masses.

The condition-specific trends observed in the present study should not be interpreted as directly transferable to other tribological configurations. Previous investigations of FDM-printed PLA, PETG, ABS, and ASA have shown that material loss and wear response depend strongly on the contact mode, printing strategy, surface condition, and post-processing route [14,15,16,21,22,23,24]. Recent studies have also shown that surface texture can influence the mechanical properties of additively manufactured PLA [25], while heat treatment can modify the tensile strength of FDM-printed PETG [26]. Therefore, the present results primarily demonstrate the relative responses of the five investigated filament systems when evaluated using the same wet–abrasive grinding protocol. The laboratory-produced PLA–1 wt.% Fe filament exhibited greater material loss than the commercial neat PLA under the selected conditions. Because the two filament systems differed not only in Fe content but also in filament-production history, polymer grade, and potentially other formulation-related factors, the observed difference in material loss cannot be isolated as an independent effect of Fe addition. Accordingly, the comparison between commercial neat PLA and laboratory-produced PLA–1 wt.% Fe should be interpreted as a comparison between two different filament formulations rather than as a controlled assessment of Fe reinforcement. The surface observations relevant to this difference are discussed in the following section.

The present ranking should be interpreted in the context of the strong dependence of FDM tribological behavior on test configuration and processing conditions. Previous studies have reported substantial changes in the wear response of printed polymers with variations in layer thickness, printing strategy, material formulation, and contact conditions [14,15,16,17,22,23,24,27]. The comparatively low material loss observed for PLA and PETG in the present wet–abrasive configuration therefore does not imply universal superiority over ABS or ASA. Rather, the results demonstrate that these materials respond differently when exposed to the same combination of abrasive grade, normal load, water lubrication, and rotational conditions. This condition-dependent behavior is particularly relevant for material selection, since rankings established under one tribological configuration may not be directly transferable to another.

From a durability-oriented perspective, the observed differences provide a condition-specific comparison of material loss under the present laboratory protocol. However, the results do not directly establish component service life, resource efficiency, or overall sustainability performance, which would require broader testing under application-relevant conditions.

### 3.2. Surface Morphology

#### 3.2.1. Optical Microscopy Observations

**Figure 4:**
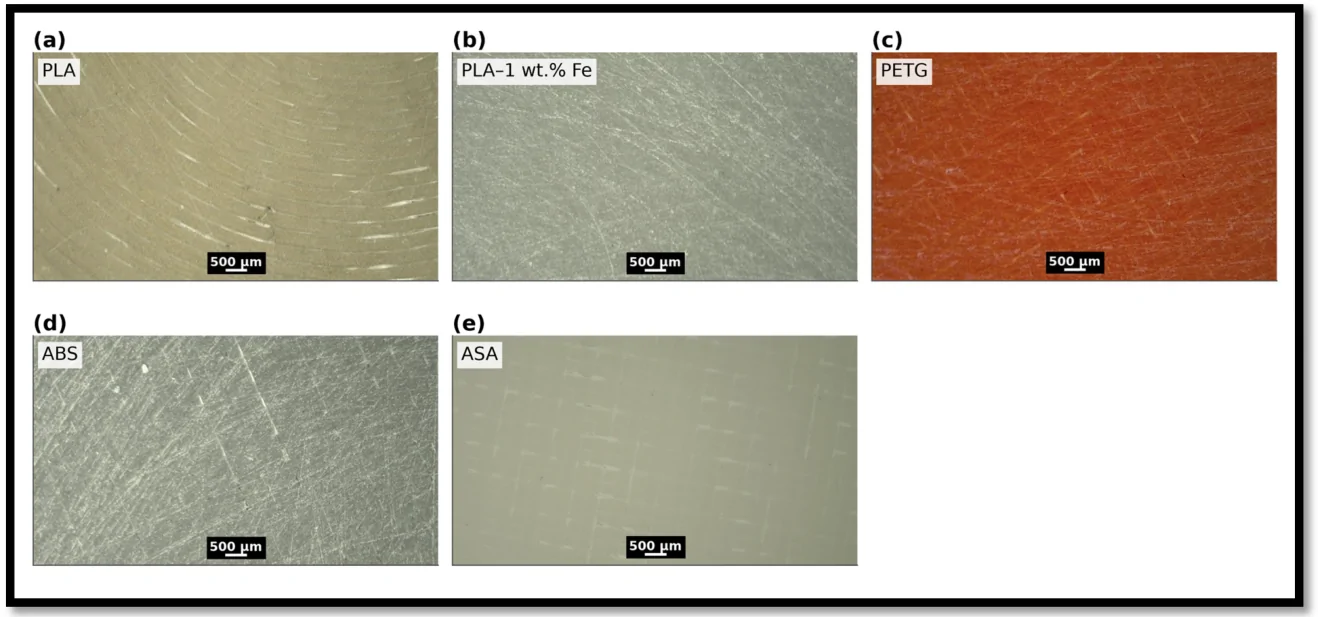
Optical images of the worn surfaces of (**a**) PLA, (**b**) PLA–1 wt.% Fe, (**c**) PETG, (**d**) ABS, and (**e**) ASA after wet abrasive grinding. All images were acquired at 40× magnification.

Representative optical micrographs of the filament surfaces after 60 min of wet–abrasive grinding are presented in Figure 4. At the applied magnification, all investigated surfaces exhibited directionally oriented abrasion marks, although differences were observed in the apparent uniformity and distribution of these features. The optical images were used for a qualitative comparison of broad surface morphology rather than for quantitative roughness assessment or definitive identification of wear mechanisms.

The worn PLA surface exhibited visible abrasion marks together with localized variations in surface contrast and topography. The PLA–1 wt.% Fe surface also showed directional marks, but its overall appearance was comparatively more heterogeneous within the observed field. PETG, ABS, and ASA showed differences in the continuity and visibility of the abrasion traces; however, the limited magnification and qualitative nature of these images do not support the assignment of specific deformation or fracture mechanisms.

The optical appearance did not vary proportionally with cumulative mass loss. In particular, ASA exhibited the greatest measured material loss despite showing a comparatively uniform surface appearance at 40× magnification. Conversely, the lower material loss of PLA was accompanied by a visibly more irregular surface texture. These observations indicate that the apparent smoothness or irregularity of a worn surface cannot be used alone as a measure of material loss. The optical images should therefore be interpreted together with the gravimetric results rather than as independent evidence of wear resistance.

#### 3.2.2. SEM and Local EDS Observations of PLA-Based Specimens

**Figure 5:**
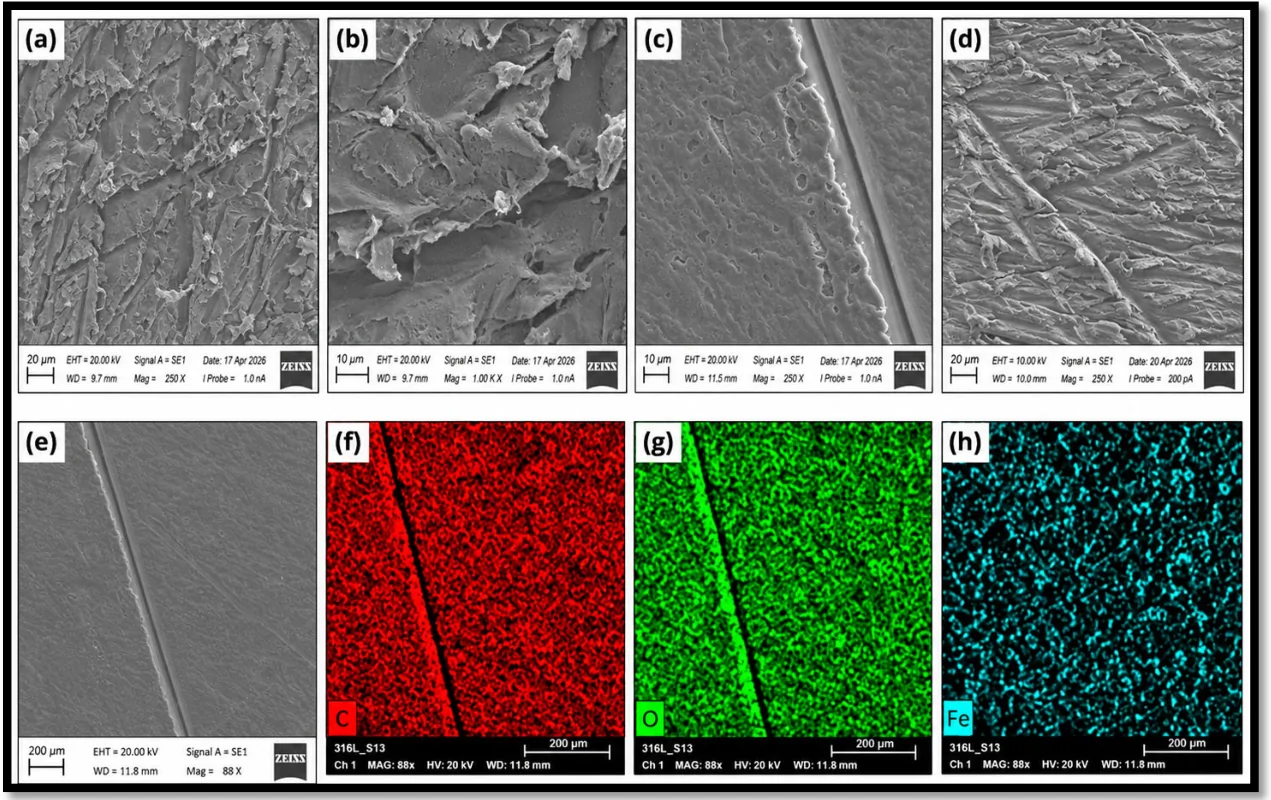
Selected SEM and EDS observations of PLA-based specimens: (**a**) worn surface of PLA–1 wt.% Fe at 250× magnification; (**b**) higher-magnification view of a representative worn region of PLA–1 wt.% Fe at 1000×; (**c**) unworn rear surface of PLA–1 wt.% Fe at 250×; and (**d**) worn surface of neat PLA at 250×. Panels (**e**–**h**) show the SEM reference image and the corresponding enlarged C, O, and Fe elemental maps obtained from a selected region of the unworn rear surface of PLA–1 wt.% Fe at 88× magnification. The higher-magnification image and elemental maps are presented as local qualitative observations and are not intended for quantitative cross-material comparison or assessment of bulk filler distribution.

Selected PLA-based surfaces were further examined by SEM to provide higher-magnification morphological information. Figure 5 presents the worn surface of PLA–1 wt.% Fe at 250× together with a higher-magnification 1000× view of a representative worn region, the unworn rear surface of PLA–1 wt.% Fe at 250×, the worn surface of neat PLA at 250×, and enlarged EDS elemental maps obtained from a selected region of the unworn rear surface of PLA–1 wt.% Fe. The 1000× image was included to provide additional visualization of local worn-surface features and was used only for qualitative interpretation rather than quantitative cross-material comparison. The worn PLA–1 wt.% Fe region exhibited an irregular topography containing elongated grooves, smeared regions, ridges, and locally disrupted surface features. The worn neat PLA surface also contained pronounced grooves and irregular surface relief, although the morphology differed from that observed for the Fe-filled formulation. In comparison, the examined unworn rear surface of PLA–1 wt.% Fe appeared relatively more uniform within the selected field.

The SEM observations demonstrate morphological differences between the selected worn regions of neat PLA and PLA–1 wt.% Fe. However, the images do not by themselves establish particle pull-out, interfacial debonding, filler agglomeration, or a unique wear mechanism. Accordingly, the present observations cannot establish the bulk Fe concentration, particle-size distribution, filler-dispersion state, degree of agglomeration, or particle–matrix interfacial characteristics within the printed filament. Therefore, the observed features are interpreted as complementary surface-level evidence rather than as proof of the internal structural origin of the higher material loss measured for PLA–1 wt.% Fe.

Local EDS measurements were performed on selected worn and unworn regions of PLA–1 wt.% Fe, and the normalized results are summarized in Table 3. The analyzed worn region was dominated by carbon and oxygen, with Fe detected at approximately 0.09 wt.%. In the analyzed region of the unworn rear surface, Fe was detected at approximately 0.72 wt.%. Thus, Fe remained locally detectable in the printed material, while the selected worn region showed a lower local Fe signal than the selected unworn region.

The lower Fe signal measured in the selected worn region should not be interpreted as direct evidence of bulk Fe depletion or preferential removal of Fe particles. Differences between the two EDS measurements may reflect local heterogeneity, the limited interaction volume of the analysis, surface coverage by the polymer-rich matrix, or differences between the selected measurement regions. Nevertheless, the combined SEM/EDS observations show that the worn surface of PLA–1 wt.% Fe remained predominantly polymer-rich within the analyzed area and did not exhibit a broadly exposed Fe-rich surface layer. Accordingly, the local SEM/EDS observations do not support the formation of a broadly exposed Fe-rich protective layer on the worn PLA–1 wt.% Fe surface under the selected grinding conditions.

### 3.3. Vickers Microhardness Response

**Figure 6:**
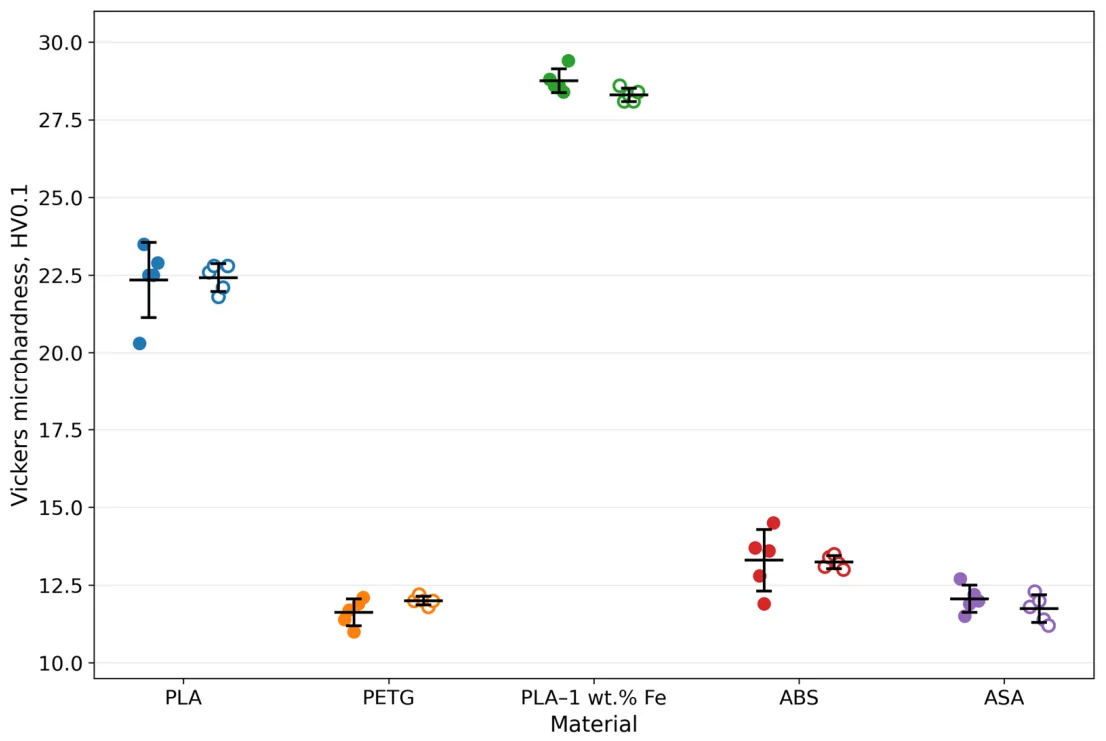
Vickers microhardness response of the investigated FDM materials measured using a common test load of HV0.1 and a dwell time of 15 s. Individual points represent five indentations performed on each of the two grinding specimens per material; the two specimens are shown separately (specimen 1, filled symbols; specimen 2, open symbols). The horizontal markers and error bars indicate the mean ± standard deviation of the five indentations of each specimen. Both specimens originated from the same print build and were not pooled.

Vickers microhardness measurements were performed on the lightly ground, unworn rear surfaces of both specimens used in the grinding experiment for each material, using a common test load of HV0.1 and a dwell time of 15 s. Five indentations were evaluated at spatially separated locations on each specimen. The individual indentation values and the corresponding specimen-level mean ± standard deviation are presented in Figure 6.

PLA–1 wt.% Fe exhibited the highest local microhardness, with specimen-level means of 28.76 ± 0.38 and 28.30 ± 0.21 HV0.1 for the two specimens, followed by neat PLA at 22.34 ± 1.21 and 22.42 ± 0.45 HV0.1. Lower values were measured for ABS (13.30 ± 0.99 and 13.24 ± 0.21 HV0.1), ASA (12.06 ± 0.44 and 11.74 ± 0.44 HV0.1), and PETG (11.62 ± 0.43 and 12.00 ± 0.14 HV0.1). The absolute difference between the two specimen means ranged from 0.06 to 0.46 HV0.1 across the five materials, with a mean absolute difference of 0.26 HV0.1. Under the common indentation condition, the descriptive hardness order was therefore:

PLA–1 wt.% Fe > PLA > ABS > ASA ≈ PETG

The relative order of ASA and PETG reversed between the two specimens, consistent with the very similar hardness values of these two materials.

PLA–1 wt.% Fe showed the highest local indentation resistance but exhibited a 60 min cumulative material loss of 22.62%, compared with 11.15% for neat PLA. Similarly, ASA and PETG displayed relatively close microhardness values, whereas their cumulative material losses were substantially different, at 50.12% and 13.00%, respectively.

These contrasting responses indicate that the material-level microhardness ranking did not directly correspond to the cumulative material-loss ranking under the present test conditions. This contrast was observed between the laboratory-produced PLA–1 wt.% Fe formulation and the commercial neat PLA; however, because the two filaments differed in production history and formulation, the contrast should not be interpreted as an isolated effect of Fe addition. A similar contrast was observed for PETG and ASA: despite their relatively close microhardness values, their 60 min cumulative material losses differed markedly. The abrasive response therefore appears to reflect the combined influence of material formulation, printed structure, deformation behavior, debris generation, local heterogeneity, and the evolving specimen–abrasive interaction. The individual contributions of these factors were not independently quantified in the present study.

The observed mismatch between microhardness and cumulative material loss indicates that the abrasive response cannot be explained by local indentation resistance alone. This observation is consistent with the classical Ratner–Lancaster framework, in which polymer abrasive response is related to a broader combination of mechanical properties rather than to hardness alone [18,19]. The optical and SEM observations provide direct evidence of grooves, irregular surface relief, smeared regions, and locally disrupted surface features in the examined areas. However, the available images do not permit definitive identification of tearing, fracture, debris-detachment, filler pull-out, or other specific material-removal mechanisms. The continued use of the same abrasive paper during each 60 min material run may also have contributed to the time-dependent response through progressive changes in abrasive cutting efficiency and debris accumulation. Therefore, the present hardness and material-loss results are best interpreted as complementary rather than directly interchangeable measures of material response.

The present material ranking also illustrates the strong condition dependence of tribological behavior in FDM polymers. Previous sliding-wear studies have reported substantially different rankings; for example, ABS has shown lower wear than PLA under ball-on-disc conditions [17], while the tribological response of ASA has been reported to vary markedly with printing parameters [16]. Together with the results of Kujawa and Ptak [20], who found no simple relationship between hardness and abrasion resistance for FDM-printed specimens, these comparisons indicate that the present material-loss response should not be attributed to a single polymer property. Rather, it appears to arise from the combined effects of material formulation, FDM-induced mesostructure, deformation behavior, and the specific fixed–abrasive wet-grinding configuration used in this study.

Microhardness was measured on the unworn rear surfaces of both specimens used in the grinding experiment for each material. Hardness data are therefore available for both grinding specimens of every material. Across the five materials, the absolute difference between the two-specimen hardness means ranged from 0.06 to 0.46 HV0.1. The order of PLA–1 wt.% Fe, PLA, and ABS was reproduced on both specimens. For ASA and PETG, the two-specimen descriptive mean hardness values were 11.90 and 11.81 HV0.1, respectively, and their relative order reversed between the two specimens. Because both specimens originated from the same print build and were ground within the same material run, this comparison represents specimen-to-specimen variation within a single build rather than build-to-build reproducibility. Taken together with the cited literature, the present results further indicate that relative wear rankings among FDM polymers can change with contact mode, lubrication condition, abrasive or counterface characteristics, applied load, and printing-related structure. Therefore, the ranking observed here should be regarded as specific to the present wet fixed–abrasive grinding configuration rather than extrapolated as a general hierarchy of PLA, PETG, ABS, and ASA [14,15,16,17,20,22,23,24,27].

### 3.4. Limitations

Several limitations should be considered when interpreting the present results. The wet–abrasive grinding experiments were performed using two physical specimens per material originating from the same print build and tested concurrently within the same material run. Therefore, build-to-build and run-to-run variability were not independently assessed. Given the limited same-build/same-run replication, the abrasive-grinding results are interpreted as exploratory and descriptive rather than as evidence of build-to-build or run-to-run reproducibility. Additional independent specimens produced and tested in separate builds and grinding runs would be required to establish broader reproducibility. Microhardness was measured on the unworn rear surfaces of both grinding specimens for each material. Hardness data are therefore available for both grinding specimens of every material. Because both specimens originated from the same print build, however, the resulting specimen-to-specimen comparison does not represent build-to-build variability, and additional specimens produced in separate builds would be required for that purpose.

The commercial neat PLA and the laboratory-produced PLA–1 wt.% Fe filament also differed in production history; therefore, their different material-loss responses cannot be attributed solely to the presence of Fe. A matched laboratory-produced neat PLA control manufactured through the same filament-processing route would be required to isolate the specific contribution of Fe addition, and the absence of such a control should therefore be considered when interpreting the PLA-based comparison. Furthermore, the same P180 SiC abrasive paper was used throughout each 60 min material run, and changes in abrasive cutting efficiency, abrasive wear or clogging, and debris accumulation were not independently quantified. These effects may therefore have contributed to the observed time-dependent material-loss rates. In addition, no specimen or interface-temperature measurements were performed during grinding. Although continuous water lubrication was used, possible localized thermal effects at the polymer–abrasive interface were not independently quantified and therefore cannot be excluded from the interpretation of the worn-surface response.

The apparent bulk densities used to estimate volumetric material loss were measured, after grinding, on the tested specimens; no unground companion specimen was available for any material. The volumetric values therefore assume that the density of the removed material equals that of the remaining material, and they are reported only as auxiliary estimates in the Supplementary Materials, while gravimetric mass loss remains the primary comparative response. In the present configuration, the platen and specimen head co-rotated, so the instantaneous relative sliding velocity resulted from the superposition of the two rotational motions and varied across the specimen contact face. A representative sliding distance could therefore not be derived reliably from the nominal specimen radius alone, and no specific wear rate was calculated. Accordingly, the results should be interpreted only for the present wet fixed–abrasive grinding configuration and should not be directly generalized to standardized abrasive-wear tests or other sliding-contact conditions. The 50.12% cumulative mass loss measured for ASA indicates substantial material removal during the test; therefore, the initial specimen geometry, effective contact area, and nominal contact-pressure condition cannot be assumed to remain unchanged during the later stages of grinding. Consequently, the calculated nominal pressure of approximately 7.1 kPa should be interpreted only as an initial loading descriptor rather than as a constant contact pressure throughout the test. The SEM/EDS observations were limited to selected regions and were not intended to verify the nominal bulk Fe concentration, quantify filler dispersion or agglomeration, or establish a unique material-removal mechanism.

## 4. Conclusions

This study compared the time-dependent abrasive wear-induced material loss, local microhardness, and worn-surface morphology of FDM-printed PLA, PETG, ABS, ASA, and PLA containing 1 wt.% Fe under a common wet–abrasive grinding protocol. After 60 min, PLA and PETG exhibited the lowest cumulative material losses, whereas ASA showed the highest. PLA–1 wt.% Fe displayed an intermediate material-loss response despite exhibiting the highest local microhardness.

A key condition-specific observation was that the microhardness ranking did not correspond directly to the cumulative material-loss trend. The laboratory-produced PLA–1 wt.% Fe showed higher microhardness than the commercial neat PLA but also substantially greater material loss, while PETG and ASA exhibited relatively similar microhardness values despite markedly different material-loss responses. These results indicate that local indentation hardness alone was insufficient to explain the observed cumulative mass-loss ranking of the investigated FDM polymers.

Optical microscopy and selected SEM/EDS observations further showed material-dependent worn-surface features and indicated local Fe detectability in the analyzed regions of the filled PLA. However, the available surface observations do not establish a unique material-removal mechanism or the bulk distribution of the Fe filler.

The findings are specific to the selected printing parameters, specimen geometry, abrasive grade, water lubrication, rotational conditions, and applied load. Accordingly, the observed differences should be interpreted as laboratory-scale, condition-specific information that may support future durability-oriented material comparisons rather than as direct evidence of service life, resource efficiency, or sustainability performance. Future studies should include larger independent specimen sets, quantified sliding distance and contact conditions, and more systematic characterization of filler distribution and particle–matrix interfaces.

## Figures and Tables

**Table 1:** Main printing parameters used for the fabrication of wet–abrasive grinding specimens.

Parameter	Unit	PLA	ABS	ASA	PETG	PLA–1 wt.% Fe
Nozzle diameter	mm	0.4	0.4	0.4	0.4	0.4
Layer height	mm	0.2	0.2	0.2	0.2	0.2
Infill	%	100	100	100	100	100
Infill pattern	—	Concentric	Concentric	Concentric	Concentric	Concentric
Wall count	—	1	1	1	1	1
Nozzle temperature	°C	220	240	240	230	220
Bed temperature	°C	60	100	100	70	60

**Table 2:** Initial mass, final mass, and cumulative mass loss after 60 min of wet–abrasive grinding.

Material	Initial Mass (g)	Final Mass (g)	Cumulative Mass Loss (%)
PLA	16.3509 ± 0.0502	14.5284 ± 0.1771	11.15 ± 0.81
PETG	17.5808 ± 0.0357	15.2960 ± 0.1113	13.00 ± 0.46
PLA–1 wt.% Fe	17.1865 ± 0.5279	13.3050 ± 0.8394	22.62 ± 2.51
ABS	14.1643 ± 0.0372	8.6021 ± 0.0494	39.27 ± 0.51
ASA	13.8891 ± 0.0875	6.9282 ± 0.2661	50.12 ± 1.60

Note: Values are reported as the mean ± standard deviation of two physical specimens originating from the same print build and tested concurrently during the same material run.

**Table 3:** Normalized EDS compositions measured in selected regions of the worn and unworn rear surfaces of PLA–1 wt.% Fe.

Surface	Carbon (wt.%)	Oxygen (wt.%)	Iron (wt.%)	Carbon (at.%)	Oxygen (at.%)	Iron (at.%)
Worn surface	53.82	46.09	0.09	60.85	39.12	0.02
Unworn rear surface	55.91	43.37	0.72	63.09	36.74	0.18

Note: The EDS results are local semi-quantitative measurements limited to the analyzed regions and should not be interpreted as representative of the bulk Fe content or overall filler distribution. Hydrogen cannot be detected by EDS; therefore, the normalized C and O values should also be interpreted as semi-quantitative. Because the specimens were Au-coated prior to SEM/EDS examination, the reported compositions were normalized only with respect to the listed C, O, and Fe signals and should not be interpreted as complete elemental compositions of the analyzed surfaces.

## Data Availability

The specimen-level mass-loss data, raw microhardness measurements, and supporting gravimetric and estimated volumetric calculations are provided in the Supplementary Excel workbook. Additional information is available from the corresponding author upon reasonable request.

## Supplementary Materials

The following supporting information can be downloaded at: https://www.mdpi.com/article/10.3390/polym18212620/s1, The following supporting information is provided in the accompanying Excel workbook: Table S1, raw Vickers microhardness measurements for the first grinding specimen; Table S2, raw Vickers microhardness measurements for the second grinding specimen; Table S3, interval-based gravimetric material-loss rates (mg/min); Table S4, post-test apparent bulk densities and estimated volumetric material loss; and Table S5, the corrected specimen-level raw mass dataset.
